# Supplementary material for: Mitochondrial complex I deficiency stratifies idiopathic Parkinson’s disease
Source: Nat Commun. 2024 Apr 29;15:3631. doi: 10.1038/s41467-024-47867-4 (PMC11059185; doi:10.1038/s41467-024-47867-4)
Supplement: Supplementary file 3 — Description of Additional Supplementary Files [file 41467_2024_47867_MOESM3_ESM.pdf]

Description of Additional Supplementary Files

File Name: Supplementary Data 1
Description: Subject demographics, pathology scores and clinical data.

File Name: Supplementary Data 2
Description: Sample experiment allocation.

File Name: Supplementary Data 3
Description: MRC IHC data.

File Name: Supplementary Data 4
Description: Data and statistics for IHC analysis of CI (NDUFS4) in 17 brain regions.

File Name: Supplementary Data 5
Description: Data and statistics for clinical and pathological parameters.

File Name: Supplementary Data 6
Description: Data and statistics for single neuron mtDNA analyses.

File Name: Supplementary Data 7
Description: Transcriptomics. Differential gene expression analysis comparing each of the nCI-PD and CI-PD groups to controls in the combined cohort. Results of all three models (Model 1-3) are shown.

File Name: Supplementary Data 8
Description: Transcriptomics. Pathway-enrichment analysis for each of the nCI-PD and CI-PD groups in the combined cohort. Results of all three models (Model 1-3) are shown.

File Name: Supplementary Data 9
Description: Single-nuclei RNA-seq. Differential gene expression analysis for each cell type, between CI-PD and controls.

File Name: Supplementary Data 10
Description: Single-nuclei RNA-seq. Differential gene expression analysis for each cell type, between nCI-PD and controls.

File Name: Supplementary Data 11
Description: Single-nuclei RNA-seq. Differential gene expression analysis for each cell type, between CI-PD and nCI-PD.

File Name: Supplementary Data 12
Description: Single-nuclei RNA-seq. Functional enrichment of differentially expressed genes for each cell type cluster by overrepresentation analysis of the Kyoto Encyclopedia of Genes and Genomes (KEGG) pathway.

File Name: Supplementary Data 13
Description: Single-nuclei RNA-seq. Overrepresentation analysis on the union of significantly differentially expressed genes in excitatory neurons, inhibitory neurons and glia.
