# Supplementary material for: Mitochondrial complex I deficiency stratifies idiopathic Parkinson’s disease
Source: Nat Commun. 2024 Apr 29;15:3631. doi: 10.1038/s41467-024-47867-4 (PMC11059185; doi:10.1038/s41467-024-47867-4)
Supplement: Supplementary file 17 — Reporting Summary [file 41467_2024_47867_MOESM17_ESM.pdf]

nature portfolio

Corresponding author(s): Charalampos Tzoulis

Last updated by author(s): 2024/02/06

Reporting Summary

Nature Portfolio wishes to improve the reproducibility of the work that we publish. This form provides structure for consistency and transparency in reporting. For further information on Nature Portfolio policies, see our Editorial Policies and the Editorial Policy Checklist.

Statistics

For all statistical analyses, confirm that the following items are present in the figure legend, table legend, main text, or Methods section.

n/a	Confirmed	
☐	☒	The exact sample size (*n*) for each experimental group/condition, given as a discrete number and unit of measurement
☐	☒	A statement on whether measurements were taken from distinct samples or whether the same sample was measured repeatedly
☐	☒	The statistical test(s) used AND whether they are one- or two-sided<br>*Only common tests should be described solely by name; describe more complex techniques in the Methods section.*
☐	☒	A description of all covariates tested
☐	☒	A description of any assumptions or corrections, such as tests of normality and adjustment for multiple comparisons
☐	☒	A full description of the statistical parameters including central tendency (e.g. means) or other basic estimates (e.g. regression coefficient) AND variation (e.g. standard deviation) or associated estimates of uncertainty (e.g. confidence intervals)
☐	☒	For null hypothesis testing, the test statistic (e.g. *F*, *t*, *r*) with confidence intervals, effect sizes, degrees of freedom and *P* value noted<br>*Give P values as exact values whenever suitable.*
☒	☐	For Bayesian analysis, information on the choice of priors and Markov chain Monte Carlo settings
☒	☐	For hierarchical and complex designs, identification of the appropriate level for tests and full reporting of outcomes
☐	☒	Estimates of effect sizes (e.g. Cohen's *d*, Pearson's *r*), indicating how they were calculated

Our web collection on statistics for biologists contains articles on many of the points above.

Software and code

Policy information about availability of computer code

Data collection	No software was used in the data collection.
Data analysis	Image analyses of the IHC data was performed in NDP.view2plus v2.7.25 (Hamamatsu). Analyses of immunohistochemistry data,c linical and demographic data and mtDNA were performed in SPSS (v28.0 .0). RNAseq analysis was performed in R v 4.l.2.

For manuscripts utilizing custom algorithms or software that are central to the research but not yet described in published literature, software must be made available to editors and reviewers. We strongly encourage code deposition in a community repository (e.g. GitHub). See the Nature Portfolio guidelines for submitting code & software for further information.

Data

Policy information about availability of data

All manuscripts must include a data availability statement. This statement should provide the following information, where applicable:

- Accession codes, unique identifiers, or web links for publicly available datasets
- A description of any restrictions on data availability
- For clinical datasets or third party data, please ensure that the statement adheres to our policy

Provide your data availability statement here.

Research involving human participants, their data, or biological material

Policy information about studies with human participants or human data. See also policy information about sex, gender (identity/presentation), and sexual orientation and race, ethnicity and racism.

Reporting on sex and gender	Our study cohort comprised 74 males and 45 females, based on assigned sex The reason for the imbalance is that Parkinson's disease is more common in males and participants had been recruited from the population. Information regarding gender was not collected in this study as it had no relevance for the research questions being examined. Sex- based analyses were conducted to compare the two iPD groups because a such analysis was of relevance to the research questions being assessed.
Reporting on race, ethnicity, or other socially relevant groupings	We analyzed biological material from a grand total of 119 individuals, comprising individuals with Parkinson"s disease (n=92) and neurologically healthy controls (n=27) from four independent cohorts (Norwegian, British, Dutch and Spanish). The mean age of the entire PD and control groups was 79+/-7.4(5D) years and 83+/-9.6(5D) years. The mean age as well as all other demographic variables and other relevant covariates of the groups used in each comparison are given in the pa per. These are toa extensive to report also in this form.
Population characteristics	The cohorts consist of controls with no known neurodegenerative disease, and individuals with pathologically confirmed Parkinson's disease. The subjects have the following mean (standard deviation) age of death: NOR Ctrl: 83(11), NOR PD 80(7), ESP Ctrl 84(9), ESP PD 78(7). In the NOR cohort, there were 4 male controls and 9 females, 26 male PD individuals and 15 females. In the ESP cohort, there were 7 male and 4 female controls, 35 male and 16 female PD individuals. A detailed overview of available clinical data is shown in Supplementary table 1.
Recruitment	Participants were not recruited specifically for this study. We used biological material (brain tissue) from the Park West population-based prospective cohort, which has been extensively published, and the Barcelona Brain Bank, the Netherlands Brain Bank and the London neurodegenerative Brain Bank.
Ethics oversight	The Regional Committee for Medical and Health Research Ethics, Western Norway (REK 2017/2082, 2010/1700, 2016/1592).

Note that full information on the approval of the study protocol must also be provided in the manuscript.

Field-specific reporting

Please select the one below that is the best fit for your research. If you are not sure, read the appropriate sections before making your selection.

☒ Life sciences ☐ Behavioural & social sciences ☐ Ecological, evolutionary & environmental sciences

For a reference copy of the document with all sections, see nature.com/documents/nr-reporting-summary-flat.pdf

Life sciences study design

All studies must disclose on these points even when the disclosure is negative.

Sample size	No power calculations were done. In this study we used all suitable samples that were available from aur Norwegian cohort and the Barcelona Brain Bank. Three additional controls from the Netherlands Brain Bank and the London neurodegenerative Brain Bank were included in the snRNA-seq analyses.
Data exclusions	No data was excluded
Replication	Our main findings were independently assessed in two independent cohorts from from two different populations (Norwegian and Spanish). The findings from the NOR cohort was replicateed in the ESP cohort.
Randomization	All available samples per group were included. The samples were allocated into experimental groups based on the presence of complex I deficiency. Groups were demographically matched as best as possible per comparison to control for possible covariates. Available samples were included in the different experiments.
Blinding	Investigators were blinded to groups (PD vs controls) during the visual assessment of histological sections and during the counting of positive and negative neurons in the ESP cohort. Observer 2 was blinded to groups during visual assessment of histological sections. Investigators were blinded to groups and cohorgs during the preprocessing of the RNAseq data.

Reporting for specific materials, systems and methods

We require information from authors about some types of materials, experimental systems and methods used in many studies. Here, indicate whether each material, system or method listed is relevant to your study. If you are not sure if a list item applies to your research, read the appropriate section before selecting a response.

Materials & experimental systems

n/a	Involved in the study	
☐	☒	Antibodies
☒	☐	Eukaryotic cell lines
☒	☐	Palaeontology and archaeology
☒	☐	Animals and other organisms
☒	☐	Clinical data
☒	☐	Dual use research of concern
☒	☐	Plants

Methods

n/a	Involved in the study	
☒	☐	ChIP-seq
☒	☐	Flow cytometry
☒	☐	MRI-based neuroimaging

Antibodies

Antibodies used	Primary antibodies were used against NDUFS4 (ab137064, polyclonal, Abcam), NDUFB8 (ab110242, monoclonal, isotype IgG1, , Abcam, validated in frozen IHC and tested in human samples), NDUFA9 (ab14713, monoclonal, isotype IgG1, Abcam), NDUFS1 (ab169540, Abcam), SDHA (ab14715, monoclonal, isotype IgG1, Abcam), UQCRC2 (ab14745, monoclonal, isotype IgG1,, Abcam), MTCOI (459600, monoclonal, isotype IgG2a, Thermo Fisher Scientific, validated by Cell treatment), ATP5A (ab14748, Abcam), and VDAC1 (ab14734, monoclonal, isotype IgG2b, Abcam). Antibodies against NDUFS4, NDUFA9, NDUFS1, SDHA, UQCRC2, ATP5a and VDAC1 were all validated in IHC in paraffin embedded tissues and tested in human samples according to the supplier (Abcam).
Validation	All antibodies were commercially available and validated for the species and application used in the work by the manufacturer. Antibodies against NDUFS4, NDUFA9, NDUFS1, SDHA, UQCRC2, ATP5a and VDAC1 were all validated in IHC in paraffin embedded tissues and tested in human samples according to the supplier (Abcam). NDUFB8 was validated in frozen IHC and tested in human samples. MTCOI was validated by Cell Treatment. References to validation is given by the supplier on their website. (abcam.com, thermofisher.com). All<br>antibodies were titrated to yield optimal working concentrations, starting from the manufacturer's recommendation. No further validation beyond that provided by the manufacturers was carried out.

Plants

Seed stocks	*Report on the source of all seed stocks or other plant material used. If applicable, state the seed stock centre and catalogue number. If plant specimens were collected from the field, describe the collection location, date and sampling procedures.*
Novel plant genotypes	*Describe the methods by which all novel plant genotypes were produced. This includes those generated by transgenic approaches, gene editing, chemical/radiation-based mutagenesis and hybridization. For transgenic lines, describe the transformation method, the number of independent lines analyzed and the generation upon which experiments were performed. For gene-edited lines, describe the editor used, the endogenous sequence targeted for editing, the targeting guide RNA sequence (if applicable) and how the editor was applied.*
Authentication	*Describe any authentication procedures for each seed stock used or novel genotype generated. Describe any experiments used to assess the effect of a mutation and, where applicable, how potential secondary effects (e.g. second site T-DNA insertions, mosiacism, off-target gene editing) were examined.*
